# Metabolic Reprogramming—A New Era How to Prevent and Treat Graft Versus Host Disease After Allogeneic Hematopoietic Stem Cell Transplantation Has Begun

**DOI:** 10.3389/fphar.2020.588449

**Published:** 2020-11-06

**Authors:** Reena Kumari, Senthilnathan Palaniyandi, Gerhard C. Hildebrandt

**Affiliations:** Division of Hematology and Blood and Marrow Transplantation, Markey Cancer Center, University of Kentucky, Lexington, KY, United States

**Keywords:** allogeneic hematopoietic cell transplantation, graft versus host disease, t cells, glycolysis, krebs cycle

## Abstract

Allogeneic hematopoietic stem cell transplantation (HSCT) is the solitary therapeutic therapy for many types of hematological cancers. The benefits of this procedure are challenged by graft vs. host disease (GVHD), causing significant morbidity and mortality. Recent advances in the metabolomics field have revolutionized our understanding of complex human diseases, clinical diagnostics and allow to trace the *de novo* biosynthesis of metabolites. There is growing evidence for metabolomics playing a role in different aspects of GVHD, and therefore metabolomic reprogramming presents a novel tool for this disease. Pre-transplant cytokine profiles and metabolic status of allogeneic transplant recipients is shown to be linked with a threat of acute GVHD. Immune reactions underlying the pathophysiology of GVHD involve higher proliferation and migration of immune cells to the target site, requiring shifts in energy supply and demand. Metabolic changes and reduced availability of oxygen result in tissue and cellular hypoxia which is extensive enough to trigger transcriptional and translational changes. T cells, major players in acute GVHD pathophysiology, show increased glucose uptake and glycolytic activity. Effector T (Teff) cells activated during nutrient limiting conditions *in vitro* or multiplying during GVHD *in vivo*, depend more on oxidative phosphorylation (OXPHOS) and fatty acid oxidation (FAO). Dyslipidemia, such as the increase of medium and long chain fatty and polyunsaturated acids in plasma of GVHD patients, has been observed. Sphingolipids associate with inflammatory conditions and cancer. Chronic GVHD (cGVHD) patients show reduced branched-chain amino acids (BCAAs) and increased sulfur-containing metabolites post HSCT. Microbiota-derived metabolites such as aryl hydrocarbon receptor (AhR) ligands, bile acids, plasmalogens and short chain fatty acids vary significantly and affect allogeneic immune responses during acute GVHD. Considering the multitude of possibilities, how altered metabolomics are involved in GVHD biology, multi-timepoints related and multivariable biomarker panels for prognosticating and understanding GVHD are needed. In this review, we will discuss the recent work addressing metabolomics reprogramming to control GVHD in detail.

## Introduction

**FIGURE 1:**
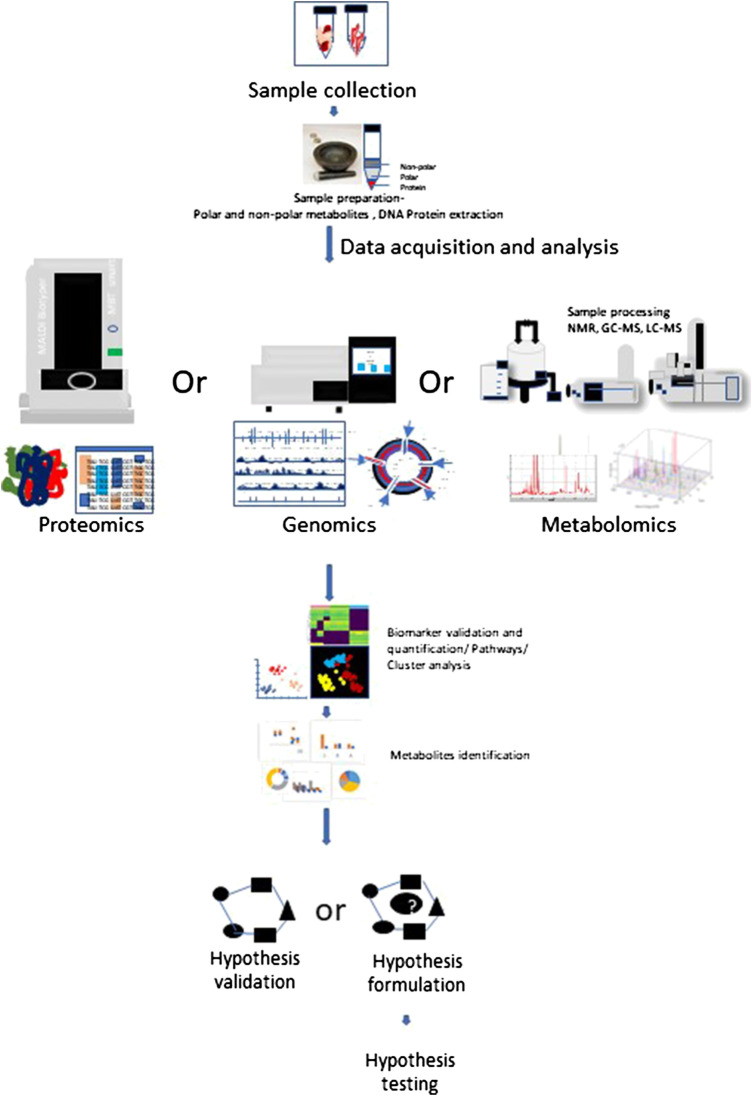
Multi-omics workflow. Metabolomics analysis involves tissue collection which needs to be snap frozen in liquid Nitrogen. Frozen tissues are minced and subjected to polar and non-polar metabolites, DNA, Protein extraction according to the requirement for target and processed further for respective approach. Collected data is subjected to metabolites detection, data reduction and analysis, which may help to generate or prove a hypothesis.

Metabolomics is an extensively used set of techniques designed to analyze metabolomic profiles in bio-fluids and tissue extracts. Metabolomics helps to understand biomarkers and phenotypic biochemical changes caused by a disease or its therapeutic intervention (Johnson et al., 2016). To reliably determine activities of metabolic pathways, use of Stable Isotope Resolved Metabolomics (SIRM) tracer approach is extensively increasing (Fan et al., 2012) (Sellers et al., 2015) (Bruntz et al., 2017) (Michonneau et al., 2019). Metabolomic analysis have shown differences between localized and systemic metabolic profiles resulted from inflammation and provide novel biomarker for therapeutic intervention in various human diseases (Kapoor et al., 2013). Therapeutic modulation of cell signaling by prioritizing alternative metabolic pathways may serve as therapeutic intervention. Figure 1 depicts the multi-omics workflow to analyze the immunoregulatory metabolites. Various different metabolic shifts have been identified in inflammatory disorders (Kominsky et al., 2010; Kang et al., 2015).

**FIGURE 2:**
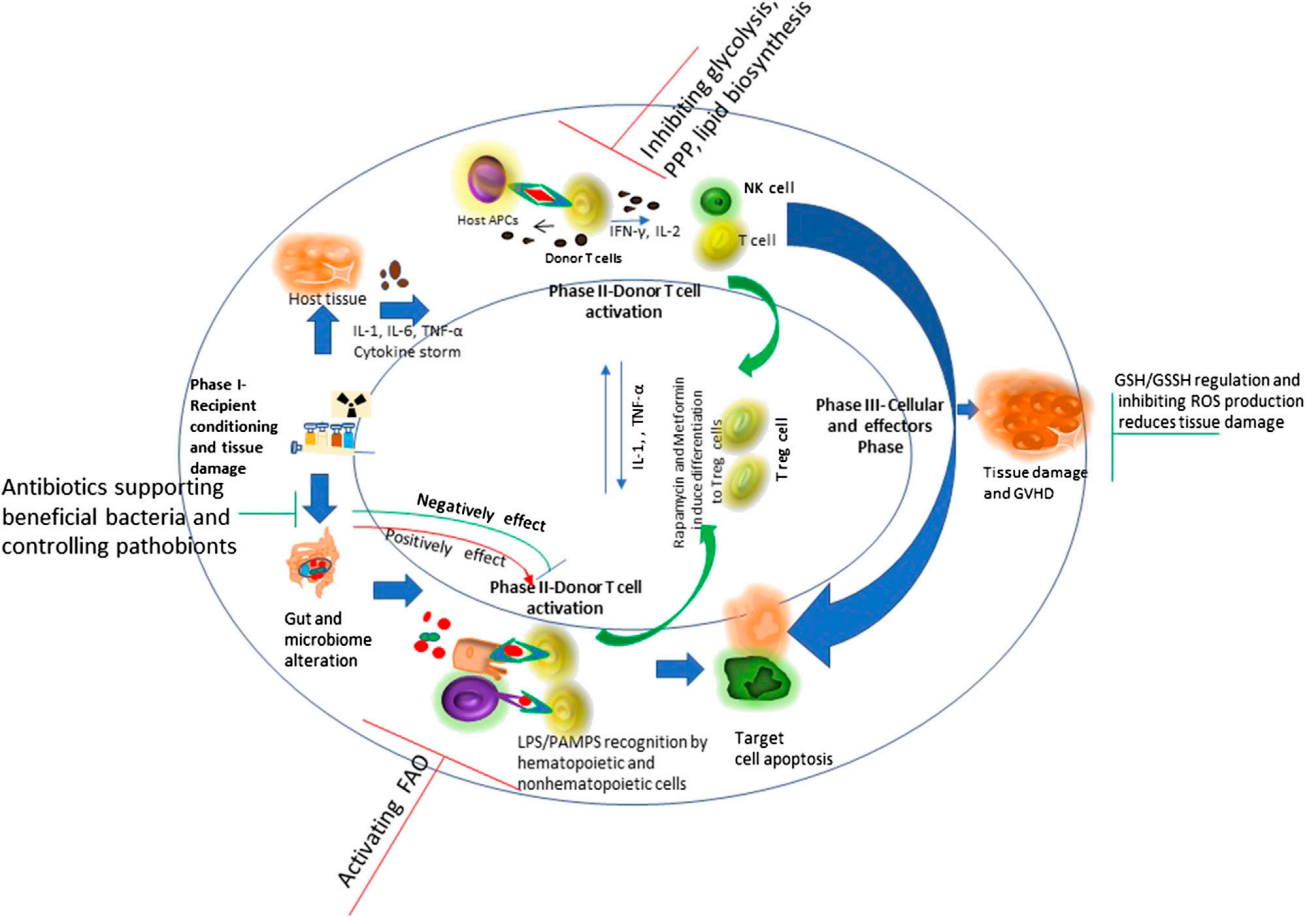
Schematic overview of graft vs. host disease (GVHD) pathophysiology with immunoregulatory aspects: Conditioning regimen at the time of transplant causes damage to the host tissues. This leads secretion of pro-inflammatory cytokines like tumor necrosis factor interleukin-6 and interleukin-1. These pro-inflammatory cytokines induce antigen presenting cell activation. Conditioning regimen also disturbs the homeostasis at intestinal mucosal Frontier due to damage caused to epithelial barrier injury and the microbiome niche. This causes increased movement of bacteria, MAMPs (microbe-associated molecular patterns), and polysaccharides to the mucosa. This consist phase-I of GVHD development. During phase 2, activated antigen presenting cells induce T cell maturation and proliferation. The effector T cells and an inflammatory cytokine storm together affect the host tissue causing further damage and perpetuating the cycle that aggravates GVHD, considering phase III. Figure modified from (Ferrara et al., 2009) Lancet 2009; 373: 1,550–61.

Allogeneic hematopoietic cell transplantation (allo-HSCT) is a potential therapeutic course of action for patients with a diversity of acquired and inherited malignant and nonmalignant disorders to establish marrow and immune function. Currently there are more than 25,000 procedures performed annually and increasing regularly. Allo-HSCT involves the infusion of donor hematopoietic stem and progenitor cells into the patient. Interactions between the donor immune system and the recipient tissue result in a devastating complicated disease, called graft vs. host disease (GVHD), which is significantly linked with mortality and morbidity (Ferrara et al., 2009). The pathophysiology of acute GVHD (aGVHD) involves donor T cells and inflammatory cytokine-mediated injury to patient’s organ tissues as a result of transplant and conditioning regimen. The primary organs affected during GVHD are intestine, lung, liver, skin and the immune system. GVHD target organs show differential kinetic and often discordant progression and vary in cytokine profiles among themselves, despite an allogeneic T cell response being considered the driving cause (Kumar et al., 2017). Figure 2 shows the phases of GVHD pathophysiology with immunoregulatory aspects -modified from Ferrara et al., 2009.

Metabolic regulation is important for immunoregulation and GVHD, and pretransplant cytokine profiles and metabolic status of allotransplant recipients are shown to be associated with a risk of later a GVHD development (Reikvam et al., 2016). Previous studies suggest differences in immune cells metabolism and may have significant association with GVHD pathology (Kalaeva et al., 2019).

Scientific reports suggest a valid possibility of inhibiting glycolysis to treat GVHD while not affecting the graft-versus- tumor (GVT) effect after allo-HSCT. Inhibition of Hexokinase-2, a glucose-metabolizing enzyme reduced activation and function of allogeneic T cells. Lower levels of glycolysis would support the generation of long-lived CD8 T cells which are important in maintaining the GVT effect (Nguyen et al., 2018).

Michoneau D. *et al* compared the metabolomic profiles of allogeneic-HSCT recipients without GVHD with healthy subjects (plasma of sibling donors obtained before stem cell collection) and observed a significant increase in the amounts fatty acid, mono and diacylglycerol and primary bile acids, which are complex lipid metabolites. Tryptophan-metabolites and taurine production were robustly decreased after transplantation, although polyamine metabolites (5-methylthioadenosine (MTA) and *N*-acetylputrescine) were increased in recipients without GVHD (Michonneau et al., 2019). These reports suggest identification and characterization of specific metabolic pathways are crucial steps in the identification of novel targets for prevention and therapy of GVHD after allo-HSCT.

## Organ and Serum Metabolomics

Previous work has shown that T effector cells (Teff) cells may show reprogramming of metabolic phenotypes induced by varying levels of oxygen tension among tissues. These changes in metabolic phenotypes are regulated by hypoxia-inducible factors (HIFs) (McNamee et al., 2013). Studies showed that hypoxia occurs concomitantly with inflammatory responses. Hypoxia can induce inflammation, and inflamed lesions often subsequently become severely hypoxic (Eltzschig and Carmeliet, 2011; Bartels et al., 2013). Under hypoxic conditions, T cells utilize glutamine in lieu of glucose for lipogenesis and may shift from oxidative to reductive metabolism (Pearce and Pearce, 2013; Nguyen et al., 2018). No studies have been conducted on whole tissue to study their metabolomics reprograming during GVHD pathophysiology. Allotransplant recipients with chronic GVHD (cGVHD) showed an altered serum metabolic profile after one year of transplant which was due to the underlying disease and related immunosuppressive treatment (Reikvam et al., 2017).

## T Cells Metabolism and Allo-HSCT

Immune cells metabolism have been shown to differ during GVHD pathophysiology and associate significantly (Kalaeva et al., 2019). Various metabolic phenotypes have been identified in inflammatory disorders (Kominsky et al., 2010; Kang et al., 2015). Metabolic switching in T cells is dynamic and is linked to functional changes on activation (van der Windt and Pearce, 2012).

Naïve T cells solely depend on oxidative phosphorylation (OXPHOS) to meet their energy requirements (Gerriets and Rathmell, 2012). Upon antigen recognition, naïve T cells differentiate into Teff cells and most of these Teff cells die, however a subset of long-lived memory T cells (Tm) sustain after antigen clearance.

Metabolism of Teff shows shift to anaerobic glycolysis as a main energy source (Gerriets and Rathmell, 2012; Yang et al., 2013). Anaerobic glycolysis produces only up to 2 mol adenosine triphosphate (ATP) per mol glucose oxidized into pyruvate and thence lactate via the mitochondrion-independent metabolism. However, OXPHOS may produce up to 30 ATP per glucose molecule. Anaerobic glycolysis supplies metabolites for cell growth and proliferation, and subsequently stimulate the pentose phosphate pathway (PPP), which builds nucleotides and amino acids that eventually produce NADPH to maintain cellular redox balance (NADP^+^/NADPH) (Vander Heiden et al., 2009). Teff also use glutamine as a carbon source to fuel the tricarboxylic acid (TCA) cycle via *α*-ketoglutarate (α-KG) through the process of glutaminolysis (Metallo et al., 2011; Wang et al., 2011). Glutamine has been considered a crucial source of energy and macromolecule production in activated T cells and their development after allo-HSCT and during GVHD (Tijaro-Ovalle et al., 2019).

Quiescent T cells (i.e., naive or memory T cells) follow a catabolic metabolism where nutrients are broken down to support cell survival. On the contrary, activated T cells acquire an anabolic metabolism, where nutrients are used to form the molecular building blocks that are integrated into cellular biomass to continue proliferation. The balance of catabolic and anabolic reactions in a cell decides the amount of ATP generated vs. consumed (van der Windt and Pearce, 2012). T cells increase their oxygen consumption compared to resting T cells and show a hyperpolarized mitochondrial membrane potential, with a simultaneous increase in reactive oxygen species (ROS) production. It has been shown that inhibition of the mitochondrial F1F0-ATPase avert GVHD without altering homeostatic reconstitution, thus OXPHOS is required for allo-reactive T cell survival (Chiaranunt et al., 2015; Tijaro-Ovalle et al., 2019). These metabolic changes in T cells on activation suggests their adjustment to new requirements and conditions and regulation of these metabolic changes may serve as innovative therapeutic approach.

T cells in aGVHD patients have been shown to be polarized toward pro-inflammatory T cells and have higher glycolytic activity in contrast to T cells of non-aGVHD patients. Importantly, *in vitro* treatment of T cells derived from aGVHD patients with the glycolysis inhibitor 3PO improved their activity through reducing the glycolytic activity (Wen, 2019). Inhibition of mTOR with rapamycin reduces glycolysis and intensify fatty acid oxidation (FAO) in donor T cells which may reduce alloreactive T cells and enhance regulatory T cell (Treg) function (Herrero-Sánchez et al., 2016).

Activated B cells share a few metabolic phenotype with T cells and show increased glucose uptake and induction of glycolysis (Doughty et al., 2006; Dufort et al., 2007).

Assmann et al. showed that higher glycolytic activity diagnosed by hyperpolarized 13C-pyruvate MRI of the liver showing high conversion of pyruvate to lactate, could differentiate allogeneic from syngeneic HSCT recipients, before chronic GVHD developed clearly. Authors observed similar metabolic changes on single cell sequencing of T cells obtained from patients undergoing allogeneic HSCT. Their finding indicated the value for using this imaging technique in the clinical post-HSCT setting which may allow early, non-invasive diagnosis of GVHD (Assmann et al., 2020).

The naïve T cells expanding into Teff cells can alternatively obtain a Treg phenotype. *In vitro* differentiated Tregs show reduced glycolytic activity and depend more on FAO and OXPHOS compared to Teff cells. Hence general metabolic phenotype of Tregs resembles to that of naïve T cells. The type 1 regulatory T cell subset (Tr1) show metabolic phenotype similar to Teff cells, showing a high rate of aerobic glycolysis. Tr1 cells reduce the T cell responses by the generating interleukin (IL)-10 and do not show Foxp3 expression (Almeida et al., 2016).

Effector function of T cells like proinflammatory cytokine production requires aerobic glycolysis, however it is downregulated along with glutamine metabolism during their transition to memory phase. In contrary FAO is induced to help memory T cell function (Zou and Chen, 2020).

It has been revealed that in naive T cells and memory T cells, Adenosine monophosphate-activated protein kinase (AMPK)-mediated oxidative metabolic state plays important role in cell survival and adapting to the energetic needs (Zou and Chen, 2020). Rational for AMPK mediated oxidative metabolism has been shown by administration of metformin. Metformin activates AMPK therefore promotes FAO and might reduce GVHD by supporting the differentiation of Treg and affecting the balance between T helper (Th)-17 and Treg cells (Park et al., 2016).

**FIGURE 3:**
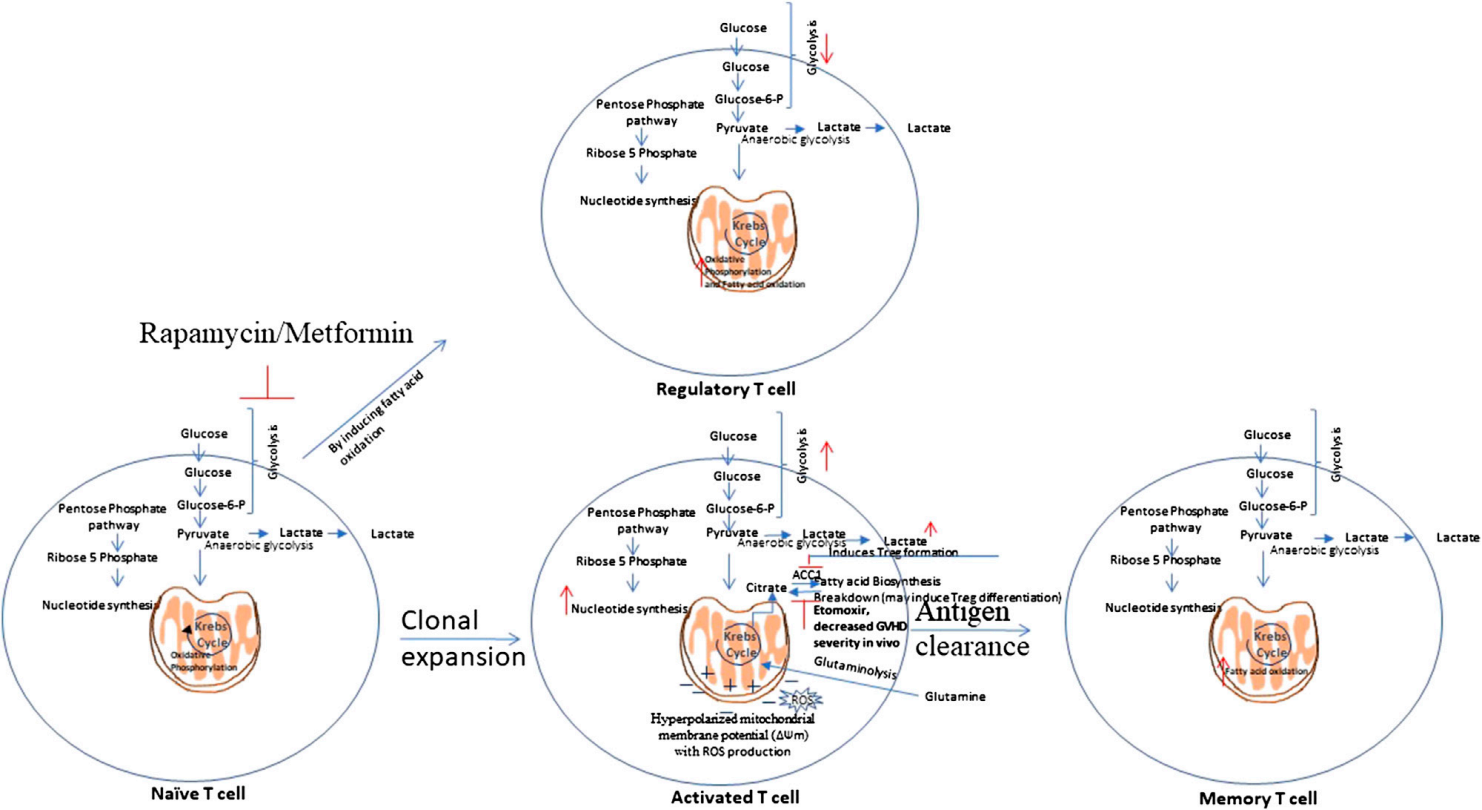
T cells metabolic reprogramming. Naïve T cells are highly dependent on Oxidative Phosphorylation (OXPHOS) and utilize catabolic metabolism which involves breaking down metabolites into smaller units that are either metabolized to produce energy or utilized in anabolic reactions. Upon antigen recognition, naïve T cells differentiate into Teff cells and most of these Teff cells die upon antigen clearance, but a subset of long-lived memory T cells (Tm) sustain. Teff cells show metabolic shift to glycolysis and show increased pentose phosphate pathway activity. Teff cells tend to utilize glutamine instead of glucose as a major lipogenic precursor. Teff cells show hyperpolarized mitochondrial membrane potential, with a simultaneous rise in the production of reactive oxygen species (ROS) that further mediate damage and inflammation. Tm cells show increased fatty acid oxidation. Rapamycin, which inhibits glycolysis, and Metformin which induces AMPK activity, that is involved in glycolysis inhibition, have shown to attenuate GVHD. This is mediated by enhanced fatty acid oxidation (FAO) in donor T cells which may reduce alloreactivity of T cells and enhance regulatory T cell (Treg) function.

Scientists suggest the necessity of novel approaches that selectively target alloreactive T cells as the approaches known to inhibit the T cell response are not specific and inhibits alloreactive and protective T cells as well. Exploring the unique metabolic profiles of activated T cells could allow one to target and inhibit them in specific manner (Zou and Chen, 2020). T cells metabolic reprogramming during their activation and differentiation is shown in Figure 3.

## Other Immune Cells Metabolism

**FIGURE 4:**
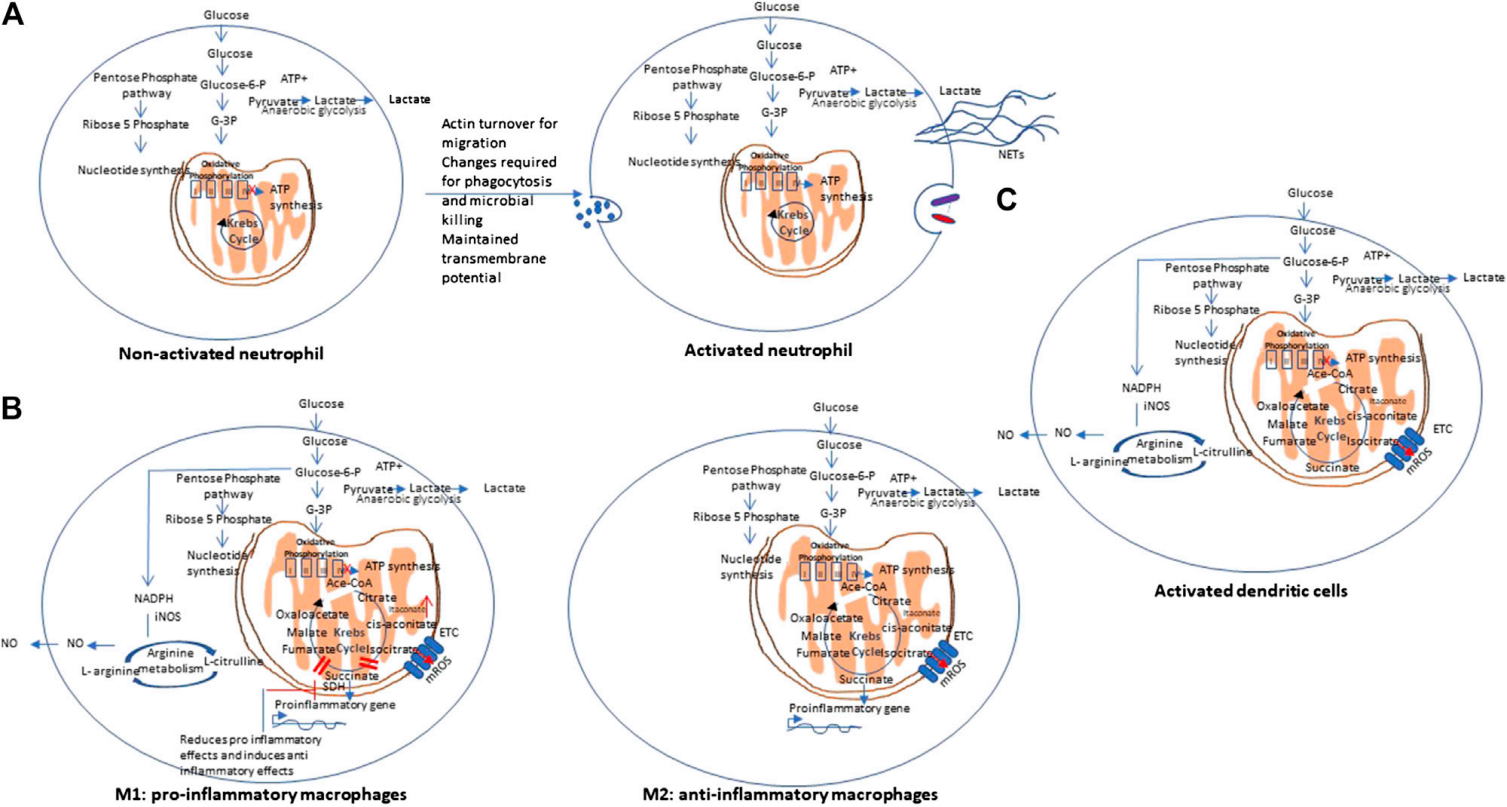
Metabolic pathways of other immune cells. **(A)** Non-activated vs. activated neutrophils: Polymorphonuclear cells are mainly glycolytic in nature, and have few mitochondria, and produce low energy from respiration to ensures their function at low oxygen concentrations (even anoxia) and maintain a transmembrane potential via the glycerol-3-phosphate shuttle. NETs: Neutrophil extracellular traps. **(B)** Metabolic pathways of Macrophages: Macrophages may show differential metabolism depending on their extreme: a pro-inflammatory (M1) and an anti-inflammatory/pro-resolving (M2). M1 macrophages are dependent solely on glycolysis and present two breaks on the TCA cycle, causing collection of itaconate and succinate. In contrast, M2 cells are mainly depend on OXPHOS, and do not show any break in TCA cycle and provides the substrates for the electron transport chain (ETC). On stimulation with lipopolysaccharides (LPS) and other pathogen-associated molecular patterns, macrophages show mitochondrial collapse (meaning a decrease in ATP production) that resulted from nitric oxide (NO) production from arginine. **(C)** Metabolic pathway of dendritic cells. Dendritic cells (DCs), on stimulation, show decreased OXPHOS, with simultaneous increase in glycolysis and pentose phosphate pathway activity, similar to M1 macrophages. On activation with lipopolysaccharides (LPS) and other pathogen-associated molecular patterns, both DCs also show mitochondrial collapse (meaning a decrease in ATP production) that resulted from nitric oxide (NO) production from arginine.

In contrast to lymphocytes that proliferate within tissues, Polymorphonuclear leukocytes (PMNs) (neutrophils), macrophages, and dendritic cells, circulate in the bloodstream and are recruited to sites of inflammation during immune response to foreign invaders. During their movement, these cells undergo various changes as they require huge amounts of energy in the form of ATP for actin turnover and migration. Further metabolic changes are needed to perform phagocytosis and microbial killing. PMNs are able to perform at deep inflammatory lesions that usually have low oxygen concentrations (even anoxia) and this is ensured by their primarily glycolytic nature and few mitochondria, and little energy production from respiration. It has been shown that PMNs have unique mitochondrial properties to maintain a transmembrane potential. This is maintained by the glycerol-3-phosphate shuttle that helps to regulate aerobic glycolysis as opposed to producing energy. PMNs develop these unique mitochondrial phenotype during their differentiation from myeloid precursor cells (Kominsky et al., 2010). Figure 4 A shows the metabolic pathway of activated neutrophils. Cytokines and cytotoxic molecules such as reactive oxygen species and reactive nitrogen species generated from macrophages and neutrophils are required to kill invading organisms and are involved in clearing infection and repairing tissue damage. These processes consume enormous amount of oxygen, ATP and NADPH and thus leads to significant metabolic changes (Kapoor et al., 2013). Critical metabolic changes and distinct phenotypes have been identified that are required for neutrophil differentiation and functions (Kumar and Dikshit, 2019; (Viola et al., 2019).

Macrophages may show differential metabolism depending on their extreme: a pro-inflammatory (M1) and an anti-inflammatory/pro-resolving (M2). M1 macrophages depend solely on glycolysis and present two stops on the TCA cycle that result in collection of itaconate and succinate. Itaconate is a microbicide compound and succinate has been considered to be involved various immune responses. In contrast, M2 cells are mainly dependent on OXPHOS, and do not show any break in TCA cycle that provides the substrates for the complexes of the electron transport chain (ETC) (Viola et al., 2019). Figure 4B represents the metabolic pathways of M1 and M2 macrophages.

Dendritic cells (DCs), on stimulation, show decreased OXPHOS, with simultaneous increase in glycolysis and pentose phosphate pathway activity, similar to M1 macrophages. Upon stimulation with lipopolysaccharides (LPS) and other pathogen-associated molecular patterns (PAMPs), both DCs and macrophages show mitochondrial collapse (meaning a decrease in ATP production) resulted from nitric oxide (NO) production from arginine (Mills et al., 2017). Figure 4C represents the metabolic pathways of activated dendritic cells.

## Possible Metabolic Biomarkers/Pathways as Therapeutic Targets

Allo-HSCT recipients developing aGVHD manifest alteration of preconditioning/pretransplant levels of various immunoregulatory metabolites. This observation suggests that these altered metabolite may serve as biomarkers for GVHD prediction (Reikvam et al., 2016).

### Glycolytic Pathway Metabolites

Energy or ATP generation in cells involves fundamental cellular processes such as Glycolysis and OXPHOS (Buck et al., 2015; Cameron et al., 2016). Glycolysis interconnects with Kreb’s cycle and the PPP, and any changes in glycolytic activity consequently affect PPP pathway, which is critical for nucleotide biogenesis, glutathione reduction, and NADPH regeneration (Filosa et al., 2003). Studies have shown that glycolysis is required for appropriate action of alloreactive T cells and GVHD development. This is evidenced by inhibition of glycolysis by targeting mTORC1 or 6-phosphofructo-2-kinase/fructose-2,6-biphosphatase 3 (PFKFB3) that reduced GVHD mortality and morbidity in mouse models (Nguyen et al., 2016; Nguyen et al., 2018). This observation suggested that glycolysis may be a possible therapeutic target to reduce GVHD development.

### Krebs Cycle Intermediates

Krebs cycle intermediates such as succinate, fumarate and citrate succinate are involved in physiological reaction related to immunity and inflammation, considering both innate and adaptive immune cells (Mills et al., 2017). These metabolites are accumulated in M1 macrophages due to presence of several break points in the Krebs cycle. The break point at the enzyme succinate dehydrogenase (SDH), that converts succinate to fumarate and also serves as complex II of the ETC, leads to collection of succinate. Oxidation of succinate by SDH results in ROS production from complex I during reverse electron transport (RET) and causes activation of HIF-1α and HIF-1α-dependent gene expression; like gene encoding IL-1β. Succinate is also known to limit the production of anti-inflammatory cytokines, particularly of IL-10. Inhibition of SDH with dimethylmalonate (DMM) inhibits production of LPS-induced mitochondrial ROS and IL-1β and various proinflammatory genes in macrophages, and enhance the expression of IL-10 and anti-inflammatory genes (Mills et al., 2017).

Macrophages use the metabolite itaconate to control excessive ROS production and hyperinflammation by which they can limit SDH function and proinflammatory response. The amount of itaconate is increased in LPS-activated M1 macrophages because of reduced expression of isocitrate dehydrogenase, as this enzyme distracts citrate away from itaconate. M1 macrophages and LPS-induced macrophages show increased expression of the enzyme Irg1, that carry out the decarboxylation of aconitate (produced from citrate) to Itaconate (Tannahill et al., 2013; Lampropoulou et al., 2016). Itaconate treatment has been shown to have anti-inflammatory effects and antibacterial effects by reducing the level of IL-1β, IL-12p70 and IL-6, as well as **i**nducible Nitric Oxide synthase (iNOS) expression and ROS production, on LPS-induction, by limiting RET as a result of decreased succinate oxidation by SDH in macrophages (Chouchani et al., 2014).

The decreased amount of isocitrate dehydrogenase results in accumulation of itaconate and citrate as well, which initiates an inflammatory response in M1 macrophages (Tannahill et al., 2013). Citrate is required for fatty acid biosynthesis, which are shown to be involved in inflammation and GVHD pathophysiology to some extent (Mills et al., 2017; Nguyen et al., 2018).

Histone acetylation regulates transcriptional activation, histone acetyltransferase enzymes acetylate amino- terminus of histone H3 and H4. Histone deacetylase (HDAC) inhibitors can result in altered pattern of gene expression and may show anti-inflammatory and immunoregulatory effects (Choi and Reddy, 2011; Choi and Reddy, 2014).

Activity of some of the enzymes regulating histone and DNA demethylation, for example the *α*-KG-dependent Jumonji C-domain-containing histone demethylases (JMJDs) and the Ten-eleven translocation (TET) family of 5mC hydroxylases, can be based on the ratio of *α*-ketoglutarate (α-KG) to succinate and this ratio can play important role to remodel the epigenome (Benit et al., 2014).

### Pentose Phosphate Pathway (PPP) Intermediates

In murine models of GVHD, alloantigen-activated T cells indicate increased PPP activity (Nguyen et al., 2016). Intracellular glucose metabolized by various reaction to Ribose-5P and is controlled by glucose-6-phosphate dehydrogenase in the oxidative branch of the PPP, which is potentially higher in allogeneic T cells (Nguyen et al., 2016). Reductive biosynthesis of antioxidant molecules such as GSH requires formation of NADPH, which is produced in the oxidative arm of the PPP. GSH is known to promote T-cell expansion by driving glycolysis and glutaminolysis, and assisting mTORC1 and c-Myc signaling during inflammation (Nguyen et al., 2018). Nguyen HD et al. observed that inhibition of FAO by Etomoxir (Eto; a CPT1A inhibitor) or inhibition of PPP by dehydroepiandrosterone (DHEA; a glucose-6 phosphate dehydrogenase inhibitor) were not enough to significantly affect donor T cell proliferation (Wang et al., 2011; Nguyen et al., 2016). However Byesdorfer et al. reported that *in vivo* inhibition of FAO by etomoxir exclusively reduced alloreactive T cells and decreased GVHD severity without affecting homeostatic T cells or immune reconstitution (Byersdorfer et al., 2013).

### Amino Acids Metabolites

Amino acid metabolism is shown to be linked with inflammation (Durham et al., 2009; Suh et al., 2014). It is shown that the branched-chain amino acids (BCAAs) leucine and isoleucine were decreased. In contrast the sulfur-containing metabolite (cystine) was raised at day +10 and day +100 in cGVHD patients when compared to cGVHD-free patients at respective time points. Group segregation analysis by hierarchical clustering to generate a heatmap on the basis of these variables clustered cGVHD patients from cGVHD-free patients (Alborghetti et al., 2019). This suggested significant involvement of BCAAs and the sulfur-containing metabolite in cGVHD pathophysiology. Another study reported altered pretransplant amino acid metabolites in plasma of patients which later developed aGVHD. This alteration involved immunoregulatory branched chain amino acids (leucine, isoleucine and valine); and proinflammatory tyrosine metabolites (p-cresol sulfate, 3-phenylpropionate) produced by the gastrointestinal microbial flora (Reikvam et al., 2017).

p-Cresol is soaked up from the intestine and detoxified in the liver by conjugation (sulfatation and glucuronidation), producing *p*-cresylsulfate and *p*-cresylglucuronide (de Loor et al., 2005) in serum. These bioproducts have proinflammatory and proapoptotic effects as they activate production of free radical from leukocyte (Poveda et al., 2014), and affect the response of endothelium to inflammatory cytokines and the cytokine release by monocytes (Schepers et al., 2007).

Tryptophan metabolism plays a significant role in immunoregulation. For example, indoleamine 2,3-dioxygenase, the rate-limiting enzyme of tryptophan degradation in the kynurenine pathway, acts in a potent immune regulatory loop and participate in the GVHD pathophysiology (Paczesny, 2013).

Patients with cGVHD showed a significantly higher levels of phenylacetate, 3-(4-hydroxyphenyl) lactate, phenylalanine, and tyramine *o*-sulfate in comparison to patients without cGVHD which could result from altered intestinal microbiota or functions. cGVHD patients in the same study also showed higher levels of biomarkers for proteolysis and accelerated protein catabolism, involving *N*-acetylserine, *N*-acetylaspartate, *N*-acetylasparagine, *N*-acetylglutamate, and 1-methylhistidine (Reikvam et al., 2017).

Significant interaction of host and microbial metabolism is seen, and the microbiome has been shown to be capable of metabolizing drugs and thus modulating host response. Production of bioactive indole-containing metabolites such as indoxyl sulfate and the antioxidant indole-3-propionic acid (derived from tryptophan metabolism) and multiple organic acids containing phenyl groups are impacted by presence of gut microbes and composition (Wikoff et al., 2009).

### Oxidative Stress and Redox Metabolism

The preceding disease conditions and the necessity for conditioning therapy for allo-HSCT may result in oxidative stress. The increased level of intracellular levels of ROS can destruct lipids, proteins and DNA and has been linked to various disease pathologies (Schieber and Chandel, 2014). The cellular ROS could contribute to GVHD pathogenicity significantly. Thioredoxin (Trx) an enzyme that is ubiquitously expressed enzyme, scavenges ROS and regulates other enzymes that metabolize H_2_O_2_ to prevent oxidative stress. Tregs express and secrete increased levels of Trx compared to conventional CD4 T cells. Trx-1 is potentially required for the resilience of Tregs to oxidative stress and encourage the expression of surface thiols in higher amount. The role of Trx is well known in controlling the expansion and/or migration of T cells (Sofi et al., 2017).

A study conducted by Reikvam and his team suggested altered protein metabolism associated with disturbed redox homeostasis in cGVHD patients, and hierarchical clustering analyses for “oxidative stress” metabolites resulted in two main clusters with high frequency of cGVHD patients in subset showing high levels of these metabolites. These metabolites increased in cGVHD patients were gamma-glutamyl amino acids (e.g., gamma-glutamylglutamate, gamma-glutamyltryptophan, gamma-glutamylphenylalanine, and gamma-glutamylthreonine) and their activity that is critical for recycling and regeneration of the antioxidant glutathione and other oxidative stress markers, considering alpha-tocopherol, cysteine sulfonic acid, and methionine sulfoxide, were also seen in cGVHD patients (Reikvam et al., 2017).

NO production has various detrimental effects which associates with increased levels of NO synthesis via the inducible form of iNOS. Detrimental effects are mainly carried out by tissue injury due to NO mediated by direct interplay of NO with target tissues. The indirectl detrimental effects of No production take place via proinflammatory functions of NO like apoptosis in intestinal epithelial cells, killing of alveolar type II epithelial cells, mediating expression of the chemokine macrophage Inflammatory Protein-2 and hereby encouraging the movement of immune cells into tissues with increased NO production.

NO production also has beneficial effects like inhibition of P-selectin expression by platelets and neutrophils, inhibition of the activation of cyclooxygenase and the production of superoxide anion by leukocytes, inhibition of Ag presentation and T cell proliferation and immunosuppression of alveolar macrophages in the lung. The balance between the detrimental and beneficial effects in inflammatory disease settings is most likely determining the effect of NO and reactive nitrogen intermediates and may be influenced by the phase and intensity of inflammatory diseases, like GVHD and idiopathic pneumonia syndrome (Hongo et al., 2004). These observations suggested that regulation of iNOS may serve as an innovative therapeutic approach and may require extensive analysis.

The early oxidation of plasma glutathione and its oxidized form (GSH/GSSG) redox couple along with significant increase in hepatic protein oxidative damage and ROS production has been observed irrespective of radiation conditioning treatment. Authors also suggested the requirement of future studies to understand the mechanisms for these alterations and examine the importance of antioxidant intervention approaches to prevent GVHD (Suh et al., 2014).

### Lipids and Their Derivatives

Recent research advancement and attention have increased the understanding of lipid metabolism considered in the improvement of inflammation and responses at mucosal Frontier. Dyslipidemia (abnormal amount of lipids) commonly occurs post-HSCT and use of effective lipid lowering therapy in this setting suggests role of lipids metabolism in modulating graft-versus-host disease (GVHD) (Marini et al., 2015). COX-derived lipid mediators named the resolvins and the maresins have been shown to reduce human PMN trans endothelial migration, DC migration, and IL-12 production (Serhan et al., 2008; Kominsky et al., 2010).

The regulators of fatty acid uptake and FAO are known to reduce crucially after autologous or allogeneic HSCT, in comparison to resting T cells. It has been shown in both human and mouse models that inhibition of ACC1, a potential mediator for *de novo* fatty acid synthesis, reduce Th17 development supporting the formation of anti-inflammatory Foxp3+ Tregs (Berod et al., 2014; Tijaro-Ovalle et al., 2019).

In addition to upregulation of FAS associated enzymes, alloreactive T cells show a propensity for the stacking up of long-chain fatty acids. Interference of acetyl-CoA carboxylase 1 (TACC1) inhibiting the FAS halted clonal expansion of alloreactive T cells *in vitro* further suggesting the critical role of FAS to promote GVHD development (Byersdorfer et al., 2013; Raha et al., 2016; Cluxton et al., 2019).

Enhanced lipid synthesis promotes the proinflammatory Teff phenotype while lipid oxidation favors iTreg differentiation, validating the role of FAS in GVHD development (Zou and Chen, 2020).

Saturated fatty acids are known to induce inflammation in part by mimicking the actions of lipopolysaccharide (Fritsche, 2015). Tripathy D *et al* showed that increased levels of plasma free fatty acid (FFA) promote oxidative stress and has a proinflammatory effect; besides it disturbs the post-ischemic flow-mediated vasodilation of the brachial artery (Tripathy et al., 2003).

Polyunsaturated fatty acids (PUFAs) are essential to tissue homeostasis and cannot be synthesized by the body and need to be obtained through dietary sources. w-6 PUFAs leads to proinflammatory lipids, whereas v-3 PUFAs are metabolized to anti-inflammatory lipid mediators (Kominsky et al., 2010).

Analysis of lipidome and metabolome in blood samples taken prior to transplant suggested a crucial pro-inflammatory metabolic profile in patients who later developed GVHD (Contaifer et al., 2018a). Lipids containing polyunsaturated fatty acid (known as modulators of inflammation) - such as arachidonic (20:4) eicosapentaenoic (20:5) and docosahexaenoic acid (22:6) containing lipids are predictors of GVHD (Contaifer et al., 2018b). Medium and long chain fatty and polyunsaturated acid were shown to rise in plasma of GVHD patients (Michonneau et al., 2018; Michonneau et al., 2019).

Polyunsaturated fatty acids are the precursor metabolites of the eicosanoid family, such as leukotriene or prostaglandin (PG) (Calder and Grimble, 2002). Leukotriene or prostaglandin are known to be associated with generation of pro-inflammatory cytokines like interferon-γ, TNF-α, and IL-17, and gut integrity respectively. Inhibition of 5-lipoxygenase (5-LO) which reduces leukotriene B4 generation from arachidonic acid has been shown to protect mice from aGVHD (Rezende et al., 2017).

Isobutyrylcarnitine and propyonylcarnitine levels which are crucial for the transport of fatty acids and the release of immunoregulatory cytokines, were altered pretransplant samples of patients who later developed GVHD (Reikvam et al., 2016). These carnitines may also affect the trafficking of immunocompetent cells and therefore play important role in GVHD.

It is suggested that systemic steroid treatment of patients with cGVHD, alters the fatty acid/triglyceride metabolism including phospholipid, lysolipid, plasmalogen, metabolites. However several of them are also increased during inflammation (Reikvam et al., 2017) thus treatment of patients need to be considered while conducting metabolomic studies.

Sterol lipids (ST) serves as a component of membrane lipids and as hormones and signaling molecules regulate T cell function (Bach and Wachtel, 2003; Wu et al., 2016; Robinson et al., 2017). Glycerophospholipids (also called Phosphoglycerides), are vital structural ingredient of cell membranes and are crucial in many biological processes and affect membrane fluidity (Lewis and McElhaney, 2009; Han, 2016). Platelet activating factor, a member of this category has been shown to have capacity to increase platelet aggregation, reduce blood pressure, and activate inflammatory processes (Engelking, 2015). Yue Liu *et al* demonstrated that during aGVHD, glycerophospholipolytic (GPL) metabolites and enzymes were significantly altered and observed five highly connected GPL metabolites that also demonstrated an potential to predict the development of aGVHD (Liu et al., 2019).

Sphingolipids (SP) are an crucial class of lipids that play fundamental roles in cell life and play various roles in foundational phases of the acute inflammatory response and are able to induce lipotoxicity and inflammation and regulate cell death (Summers, 2006; D'Angelo et al., 2013; Taniguchi and Okazaki, 2014; Grösch et al., 2018).

Sphingolipid altered metabolism is seen in the alveolar compartment, i.e., the important lung functional unit involved in gas exchange, and is associated with inflammatory reaction and ceramide increase, especially, responsible for the shift to pathological hyperinflammation (Ghidoni et al., 2015).

Previous studies demonstrated that alloreactive Teff cells use fatty acids (FAs) as a power origin to assist their *in vivo* activation specifically during GVHD and not following acute activation (Byersdorfer et al., 2013). A recent study reported that the stearic acid/palmitic acid (SA/PA) ratio is important in the diagnosis of grade II–IV aGVHD and demonstrated that patients with high SA/PA ratios on day 7 after HSCT were unlikely to develop II–IV aGVHD compared to patients with low SA/PA ratios (Wu et al., 2018). Lipids play a diverse role in various physiological processes, therefore extensive exploration of lipidomics is required to define their mechanism in GVHD pathophysiology.

FTY720 (fingolimod) is a high-affinity agonist for four of five known Sphingosine 1-phosphate (S1P) receptors and decreases aGVHD mortality without loss of GVT effects. This effect is exerted by its immunomodulatory effects specifically by sequestering lymphocytes within secondary lymphoid organs, inhibiting circulation to peripheral sites of inflammation (Kim et al., 2003; Hashimoto et al., 2007).

Reikvam et al. reported that the presence of cGVHD was associated with significantly higher levels of (1) the three lysolipid metabolites 1-linoleoyl- GPC (18:2), 1-oleoyl-GPC (18:1), 1-palmitoleoyl-GPC (16:1), 2) the eicosanoid 12-HETE; and 3) the sphingolipid sphingosine consistent with ongoing inflammation, which could be a metabolic signature (Reikvam et al., 2017).

### Microbial Metabolites

In addition to shift in host metabolism, variation in microbiota-derived metabolites may contribute significantly to GVHD pathophysiology. The microbiota metabolome, considering the products produced by host metabolism, microbial metabolism, and mammalian–microbial co-metabolism in the intestines, affects the GVHD pathophysiology and development. Microbial metabolites like SCFA generated by microbial fermentation of dietary fibers, are able to induce H3 acetylation in the locus of Foxp3; therefore increasing the counts of Tregs directly and TGF-B production in the intestine, the recruitment of Treg, epithelial barrier protection while protecting against danger associated molecular patterns and PAMPs release and decreased apoptosis in gut, all have been shown able to mitigate GVHD. These effects of SCFA are mediated by signaling through various G protein receptors (Wong et al., 2006; Gaboriau-Routhiau et al., 2009; Mathewson et al., 2016; Pandiyan et al., 2019)).

At the onset of aGVHD, especially aryl hydrocarbon receptor (AhR) ligands, bile acids and plasmalogens has been shown to vary, which may affect the allogeneic immune response during aGVHD. The reduced production of AhR ligands by microbiota could impair indoleamine 2,3-dioxygenase (IDO) stimulation and is known to affect allogeneic T cell reactivity (Michonneau et al., 2019).

**FIGURE 5:**
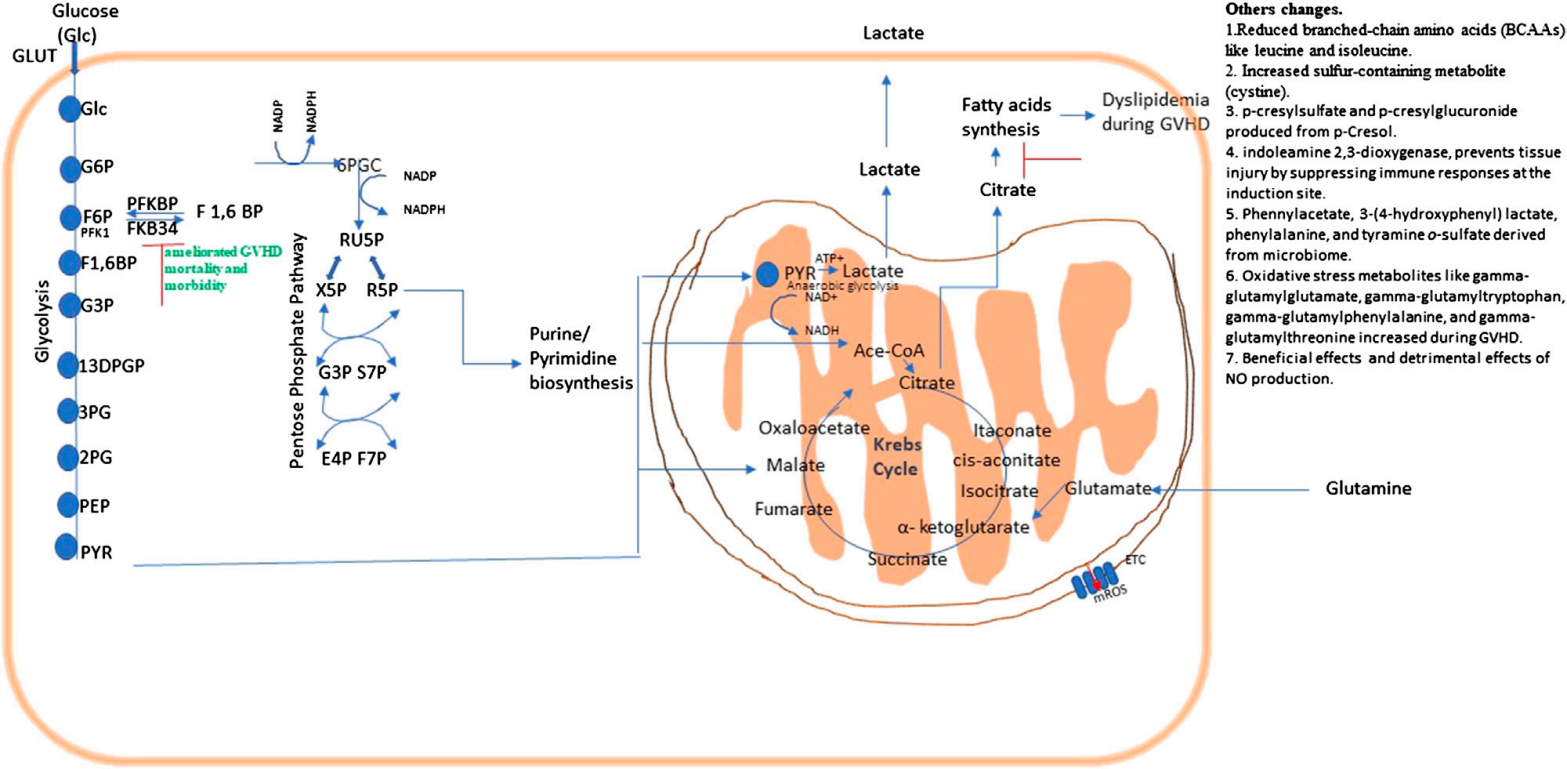
Metabolic pathways and their possible therapeutic approach in graft vs. host disease (GVHD). Energy or ATP generation in cells involve fundamental cellular processes glycolysis and OXPHOS. Glycolysis interconnects with Kreb’s cycle and the PPP, which is necessary for fundamental metabolic process, and NADPH regeneration. Metabolic changes in a pathway may consequently affects others as they are all interconnected.

IDO regulates immune metabolism by catalyzing Tryptophan (TRP) catabolism and that generates kynurenine pathway metabolites. These metabolites are biologically active, both as natural immunologically-active ligands for the AhR and by depleting TRP to trigger amino-acid sensing signal-transduction pathways, and serving as direct intracellular signaling molecule in DCs (Munn and Mellor, 2013). Jasperson et al., 2008 suggested that IDO is capable of decreasing T-cell proliferation and survival at the site of expression, thus diminished colonic inflammation and reduced GVHD severity. This suggested that modulation of the IDO pathway can prove an efficacious approach for treatment of GVHD (Jasperson et al., 2008). Another study reported that the secondary bile acid hyocholate and the primary bile acids (glycochenodeoxycholate sulfate, taurocholate, and glycocholate) were increased in patients with cGVHD (Reikvam et al., 2017). (Overview in Figure 5.)

## Summary

Literature cited on metabolic reprograming after allo-HSCT and during GVHD pathophysiology indicates the substantial involvement of metabolic changes during development and progression of GVHD and provides a growing plethora of mechanistic insights into its complex pathophysiology. The reports outlined in this review warrant the extensive analysis of GVHD target organs and immune cells metabolism involved. The relevant pathway for mitigating GVHD seems reduction of glycolysis, PPP, NO production, fatty acids biosynthesis and GSH/GSSH regulation and activation of fatty acid oxidation and tryptophan metabolites. Table 1 shows possible potential targets for GVHD treatment. Further studies in this field high likely will reveal unrecognized tissue and cell specific metabolic changes during the disease pathology proof helpful in the clinical understanding of GVHD and in the generation of novel ideas to design innovative therapeutic approaches.

**TABLE 1:** Possible potential therapeutic targets used in past or worth to try in future.

Serial. No	Possible targets	Action	Significance/background	References
1	Hexokinase-2	Reduced glycolysis	Glucose-metabolizing enzymes reduced activation and function of allogeneic T cells. Lower levels of glycolysis would support the generation of long-lived CD8 T cells which are important in maintaining the GVT effect.	(Nguyen et al., 2018)
2	Glutamine uptake by T cells and thus glutaminolysis	Inhibit lipogenesis	T cells utilize glutamine in lieu of glucose for lipogenesis and may shift from oxidative to reductive metabolism.	(Pearce and Pearce, 2013; Nguyen et al., 2018)
3	Anaerobic glycolysis	Inhibition	Metabolism of Teff shows shift to anaerobic glycolysis as a main energy source.	(Gerriets and Rathmell, 2012; Yang et al., 2013)
4	Pentose phosphate pathway (PPP)	Inhibition	In murine models of GVHD, alloantigen-activated T cells indicate increased PPP activity.	(Nguyen et al., 2016)
5	Mitochondrial F1F0-ATPase	Inhibition	Inhibition of the mitochondrial F1F0-ATPase avert GVHD without altering homeostatic reconstitution, thus OXPHOS is required for allo-reactive T cell survival.	(Chiaranunt et al., 2015; Tijaro-Ovalle et al., 2019)
6	Adenosine monophosphate activated protein kinase (AMPK)	Activation	Metformin activates AMPK therefore promotes FAO and might reduce GVHD by supporting the differentiation of Treg and affecting the balance between T helper (Th)-17 and Treg cells.	(Park et al., 2016)
7	Glycerol-3-phosphate shuttle	Inhibition	PMNs have unique mitochondrial properties to maintain a transmembrane potential. This is maintained by the glycerol-3-phosphate shuttle that helps to regulate aerobic glycolysis as opposed to producing energy.	(Kominsky et al., 2010)
8	Isocitrate dehydrogenase (IDH1)	Induction	IDH1 allows the withdrawal of citrate from the cycle. Cytosolic citrate is broken down by ATP-citrate lyase (ACLY) to oxaloacetate and acetyl-CoA. Acetyl-CoA can be used as a substrate for fatty acid synthesis.	(Williams and O’Neill, 2018)
9	Succinate dehydrogenase A (SDHA)	Induction	Allogeneic recipients (villin-Cre+SDHAfl/fl), with intestinal epithelial cell (IEC) specific SDHA KO mice, demonstrated significantly greater mortality and gastro-intestinal GVHD.	(Fujiwara et al., 2019)
10	Glutathione (GSH)	Inhibition	GSH is known to promote T-cell expansion by driving glycolysis and glutaminolysis, and assisting mTORC1 and c-Myc signaling during inflammation.	(Nguyen et al., 2018)
11	Fatty acid oxidation (FAO)	Inhibition	*In vivo* inhibition of FAO by etomoxir exclusively reduced alloreactive T cells and decreased GVHD severity without affecting homeostatic T cells or immune reconstitution.	(Byersdorfer et al., 2013)
12	Tyrosine metabolites (p-cresol sulfate, *p*-cresylsulfate and *p*-cresylglucuronide)	Inhibition	Several metabolites from the cytokine-responsive kynurenine pathway for tryptophan degradation, phenylalanine and tyrosine metabolites derived from the gut microbial flora were increased in patients with cGVHD compared to controls and shown association with inflammation.	(de Loor et al., 2005; Schepers et al., 2007; Liabeuf et al., 2013; Sun et al., 2013; Poveda et al., 2014; Reikvam et al., 2017)
13	Branched chain amino acids (BCAA)	Inhibition/activation	BCAA have been shown to increase in patients with cGVHD compared to controls, however, they have both pro and anti-inflammatory role.	(Lee et al., 2017; Reikvam et al., 2017; Zhenyukh et al., 2017)
14	Indoleamine 2,3-dioxygenase	Induction	Indoleamine 2,3-dioxygenase, the rate-limiting enzyme of tryptophan degradation in the kynurenine pathway, acts in a potent immune regulatory loop. It is suggested that IDO is capable of decreasing T-cell proliferation and survival at the site of expression, thus diminished colonic inflammation and reduced GVHD severity.	(Jasperson et al., 2008; Paczesny, 2013)
15	G-protein coupled receptor trace amine 1 (TAAR1)	Inhibition	Patients with cGVHD showed a significantly higher level of tyramine *o*-sulfate in comparison to patients without cGVHD. *Lactobacillus* species, a commensal microbe, showed association with GVHD, produce tyramine, a trace amine and a ligand for TAAR1.	(Reikvam et al., 2017; Gwilt et al., 2019)
16	Nitric oxide synthase (NOS)	Inhibition	NO production has proinflammatory functions like apoptosis in intestinal epithelial cells, killing of alveolar type II epithelial cells, mediating expression of the chemokine macrophage inflammatory Protein-2 (MIP-2) and encourage the immune cells migration with increased NO production.	(Vallance and Leiper, 2002)
17	GSH/GSSG dysregulation	Regulation	The early oxidation of plasma glutathione and its oxidized form (GSH/GSSG) redox couple along with significant increase in hepatic protein oxidative damage and ROS production has been observed irrespective of radiation conditioning treatment.	(Suh et al., 2014)
18	Resolvins and maresins	Supplementation	COX-derived lipid mediators named the resolvins and the maresins have been shown to reduce human PMN trans endothelial migration, DC migration, and IL-12 production.	(Serhan et al., 2008; Kominsky et al., 2010)
19	Acetyl-CoA carboxylase 1 (ACC1)	Inhibition	It has been shown in both human and mouse models that inhibition of ACC1, a potential mediator for *de novo* fatty acid synthesis, reduce Th17 development supporting the formation of anti-inflammatory Foxp3+ Tregs, suggesting the critical role of FAS to promote GVHD development.	(Berod et al., 2014; Tijaro-Ovalle et al., 2019)^12,26,27^
20	Lipid oxidation	Activation	Enhanced lipid synthesis promotes the proinflammatory Teff phenotype while lipid oxidation favors iTreg differentiation, validating the role of FAS in GVHD development.	(Zou and Chen, 2020)
21	v-3 PUFAs	Supplementation	Polyunsaturated fatty acids (PUFAs) are essential to tissue homeostasis and cannot be synthesized by the body and need to be obtained through dietary sources. w-6 PUFAs leads to proinflammatory lipids, whereas v-3 PUFAs are metabolized to anti-inflammatory lipid mediators.	(Kominsky et al., 2010)
22	5-Lipoxygenase (5-LO)	Inhibition	Leukotriene or prostaglandin are known to be associated with generation of pro-inflammatory cytokines like interferon-γ, TNF-α, and IL-17, and gut integrity respectively. Inhibition of 5-lipoxygenase (5-LO) which reduces leukotriene B4 generation from arachidonic acid has been shown to protect mice from aGVHD (Rezende et al., 2017).	(Rezende et al., 2017)
23	L-carnitine acyltransferases	Inhibition	L-Carnitine acyltransferases catalyze the reversible transfer of acyl groups between coenzyme A and L-carnitine, converting acyl-CoA esters into acyl-carnitine esters. Isobutyrylcarnitine and propyonylcarnitine levels which are crucial for the transport of fatty acids and the release of immunoregulatory cytokines, were higher in pretransplant samples of patients who later developed GVHD.	(Reikvam et al., 2016; Adeva-Andany et al., 2017)
24	Palmitic acid	Reduced intake	It is demonstrated that patients with high stearic acid/palmitic acid (SA/PA) ratios on day 7 after HSCT were unlikely to develop II–IV aGVHD compared to patients with low SA/PA ratios. Palmitic acid serves as TLR agonist and in the cell, this is converted into phospholipids, diacylglycerol and ceramides which trigger various signaling pathways, common for LPS-mediated TLR4 activation.	(Wu et al., 2018; Korbecki and Bajdak-Rusinek, 2019)
25	Sphingosine 1-phosphate receptor	Induction	FTY720 (fingolimod) is a high-affinity agonist for four of five known sphingosine 1-phosphate (S1P) receptors and decreases aGVHD mortality without loss of GVT effects.	(Kim et al., 2003; Hashimoto et al., 2007; Reikvam et al., 2017)

## Author Contributions

All authors listed, have made substantial, direct and conceptual contribution to the work, and approved it for publication.

## Conflict of Interest

The authors declare that the research was conducted in the absence of any commercial or financial relationships that could be construed as a potential conflict of interest.

## References

[B1] Adeva-Andany M. M. Calvo-Castro I. Fernández-Fernández C. Donapetry-García C. Pedre-Piñeiro A. M. (2017). Significance ofl-carnitine for human health. IUBMB Life 69 (8), 578–594. 10.1002/iub.1646 28653367

[B2] Alborghetti M. R. Correa M. E. P. Whangbo J. Shi X. Aricetti J. A. Silva A. A. d. (2019). Clinical metabolomics identifies blood serum branched chain amino acids as potential predictive biomarkers for chronic graft vs. Host disease. Front. Oncol. 9, 141 10.3389/fonc.2019.00141 30949447 PMC6436081

[B3] Almeida L. Lochner M. Berod L. Sparwasser T. (2016). Metabolic pathways in T cell activation and lineage differentiation. Semin. Immunol. 28 (5), 514–524. 10.1016/j.smim.2016.10.009 27825556

[B4] Assmann J. C. Farthing D. E. Saito K. Maglakelidze N. Oliver B. Warrick K. A. (2020). Glycolytic metabolism of pathogenic T cells enables early detection of GvHD by ^13^C-MRI. bioRxiv, 2020, 984609 10.1101/2020.03.16.984609 PMC7808015 32785680

[B5] Bach D. Wachtel E. (2003). Phospholipid/cholesterol model membranes: formation of cholesterol crystallites. Biochim. Biophys. Acta 1610 (2), 187–197. 10.1016/s0005-2736(03)00017-8 12648773

[B6] Bartels K. Grenz A. Eltzschig H. K. (2013). Hypoxia and inflammation are two sides of the same coin. Proc. Natl. Acad. Sci. U.S.A. 110 (46), 18351–18352. 10.1073/pnas.1318345110 24187149 PMC3831992

[B7] Bénit P. Letouzé E. Rak M. Aubry L. Burnichon N. Favier J. (2014). Unsuspected task for an old team: succinate, fumarate and other Krebs cycle acids in metabolic remodeling. Biochim. Biophys. Acta Bioenerg. 1837 (8), 1330–1337. 10.1016/j.bbabio.2014.03.013 24699309

[B8] Berod L. Friedrich C. Nandan A. Freitag J. Hagemann S. Harmrolfs K. (2014). De novo fatty acid synthesis controls the fate between regulatory T and T helper 17 cells. Nat. Med. 20 (11), 1327–1333. 10.1038/nm.3704 25282359

[B9] Bruntz R. C. Lane A. N. Higashi R. M. Fan T. W.-M. (2017). Exploring cancer metabolism using stable isotope-resolved metabolomics (SIRM). J. Biol. Chem. 292 (28), 11601–11609. 10.1074/jbc.R117.776054 28592486 PMC5512057

[B10] Buck M. D. O’Sullivan D. Pearce E. L. (2015). T cell metabolism drives immunity. J. Exp. Med. 212 (9), 1345–1360. 10.1084/jem.20151159 26261266 PMC4548052

[B11] Byersdorfer C. A. Tkachev V. Opipari A. W. Goodell S. Swanson J. Sandquist S. (2013). Effector T cells require fatty acid metabolism during murine graft-versus-host disease. Blood 122 (18), 3230–3237. 10.1182/blood-2013-04-495515 24046012 PMC3814737

[B12] Calder P. Grimble R. (2002). Polyunsaturated fatty acids, inflammation and immunity. Eur. J. Clin. Nutr. 56 (Suppl. 3), S14–S19. 10.1038/sj.ejcn.1601478 12142955

[B13] Cameron A. M. Lawless S. J. Pearce E. J. (2016). Metabolism and acetylation in innate immune cell function and fate. Semin. Immunol. 28 (5), 408–416. 10.1016/j.smim.2016.10.003 28340958 PMC10911065

[B14] Chiaranunt P. Ferrara J. L. M. Byersdorfer C. A. (2015). Rethinking the paradigm: how comparative studies on fatty acid oxidation inform our understanding of T cell metabolism. Mol. Immunol. 68 (2 Pt C), 564–574. 10.1016/j.molimm.2015.07.023 26359186 PMC11523081

[B15] Choi S. Reddy P. (2011). HDAC inhibition and graft versus host disease. Mol. Med. 17 (5–6), 404–416. 10.2119/molmed.2011.00007 21298214 PMC3105142

[B16] Choi S. W. Reddy P. (2014). Current and emerging strategies for the prevention of graft-versus-host disease. Nat. Rev. Clin. Oncol. 11 (9), 536–547. 10.1038/nrclinonc.2014.102 24958183 PMC4151470

[B17] Chouchani E. T. Pell V. R. Gaude E. Aksentijević D. Sundier S. Y. Robb E. L. (2014). Ischaemic accumulation of succinate controls reperfusion injury through mitochondrial ROS. Nature 515 (7527), 431–435. 10.1038/nature13909 25383517 PMC4255242

[B18] Cluxton D. Petrasca A. Moran B. Fletcher J. M. (2019). Differential regulation of human Treg and Th17 cells by fatty acid synthesis and glycolysis. Front. Immunol. 10, 115 10.3389/fimmu.2019.00115 30778354 PMC6369198

[B19] Contaifer D. Roberts C. H. Kumar N. G. Natarajan R. Fisher B. J. Leslie K. (2018a). Risk stratification of allogeneic stem cell recipients with respect to the potential for development of GVHD via their pre-transplant plasma lipid and metabolic signature. bioRxiv, 475244 10.1101/475244 PMC6721383 31349646

[B20] Contaifer D. Roberts C. H. Warncke U. Natarajan R. Fisher B. J. Toor A. A. (2018b). Elucidating a lipidomic and metabolomic signature of gvhd in recipients of allogeneic stem cell transplants. Biol. Blood Marrow Transplant. 24 (3), S185–S186. 10.1016/j.bbmt.2017.12.138

[B21] D'Angelo G. Capasso S. Sticco L. Russo D. (2013). Glycosphingolipids: synthesis and functions. FEBS J. 280 (24), 6338–6353. 10.1111/febs.12559 24165035

[B22] de Loor H. Bammens B. Evenepoel P. De Preter V. Verbeke K. (2005). Gas chromatographic-mass spectrometric analysis for measurement of p-cresol and its conjugated metabolites in uremic and normal serum. Clin. Chem. 51 (8), 1535–1538. 10.1373/clinchem.2005.050781 16040852

[B23] Doughty C. A. Bleiman B. F. Wagner D. J. Dufort F. J. Mataraza J. M. Roberts M. F. (2006). Antigen receptor-mediated changes in glucose metabolism in B lymphocytes: role of phosphatidylinositol 3-kinase signaling in the glycolytic control of growth. Blood 107 (11), 4458–4465. 10.1182/blood-2005-12-4788 16449529 PMC1895797

[B24] Dufort F. J. Bleiman B. F. Gumina M. R. Blair D. Wagner D. J. Roberts M. F. (2007). Cutting edge: IL-4-mediated protection of primary B lymphocytes from apoptosis via Stat6-dependent regulation of glycolytic metabolism. J. Immunol. 179 (8), 4953–4957. 10.4049/jimmunol.179.8.4953 17911579

[B25] Durham W. J. Dillon E. L. Sheffield-Moore M. (2009). Inflammatory burden and amino acid metabolism in cancer cachexia. Curr. Opin. Clin. Nutr. Metab. Care 12 (1), 72–77. 10.1097/MCO.0b013e32831cef61 19057191 PMC2742684

[B26] Eltzschig H. K. Carmeliet P. (2011). Hypoxia and inflammation. N. Engl. J. Med. 364 (7), 656–665. 10.1056/NEJMra0910283 21323543 PMC3930928

[B27] Engelking L. R. (2015). “Introduction to section III,” in Textbook of veterinary physiological chemistry. 3rd Edn Editor Engelking L. R. (Boston: Academic Press), 116.

[B28] Fan T. W.-M. Lorkiewicz P. K. Sellers K. Moseley H. N. B. Higashi R. M. Lane A. N. (2012). Stable isotope-resolved metabolomics and applications for drug development. Pharmacol. Therapeut. 133 (3), 366–391. 10.1016/j.pharmthera.2011.12.007 PMC3471671 22212615

[B29] Ferrara J. L. Levine J. E. Reddy P. Holler E. (2009). Graft-versus-host disease. Lancet 373 (9674), 1550–1561. 10.1016/s0140-6736(09)60237-3 19282026 PMC2735047

[B30] Filosa S. Fico A. Paglialunga F. Balestrieri M. Crooke A. Verde P. (2003). Failure to increase glucose consumption through the pentose-phosphate pathway results in the death of glucose-6-phosphate dehydrogenase gene-deleted mouse embryonic stem cells subjected to oxidative stress. Biochem. J. 370 (Pt 3), 935–943. 10.1042/bj20021614 12466018 PMC1223222

[B31] Fritsche K. L. (2015). The science of fatty acids and inflammation. Adv. Nutr. 6 (3), 293S–301S. 10.3945/an.114.006940 25979502 PMC4424767

[B32] Fujiwara H. Mathew A. V. Kovalenko I. Anupama P. Peltier D. Kim S. (2019). Mitochondrial complex II in intestinal epithelial cells is a critical metabolic checkpoint that regulates severity of gastrointestinal graft-versus-host disease. Blood 134 (Suppl. 1), 584–584. 10.1182/blood-2019-126775

[B33] Gaboriau-Routhiau V. Rakotobe S. Lécuyer E. Mulder I. Lan A. Bridonneau C. (2009). The key role of segmented filamentous bacteria in the coordinated maturation of gut helper T cell responses. Immunity 31 (4), 677–689. 10.1016/j.immuni.2009.08.020 19833089

[B34] Gerriets V. A. Rathmell J. C. (2012). Metabolic pathways in T cell fate and function. Trends Immunol. 33 (4), 168–173. 10.1016/j.it.2012.01.010 22342741 PMC3319512

[B35] Ghidoni R. Caretti A. Signorelli P. (2015). Role of sphingolipids in the pathobiology of lung inflammation. Mediat. Inflamm. 2015, 1 10.1155/2015/487508 PMC4681829 26770018

[B36] Grösch S. Alessenko A. V. Albi E. (2018). The many facets of sphingolipids in the specific phases of acute inflammatory response. Mediat. Inflamm. 2018, 5378284 10.1155/2018/5378284. PMC5818902 29540995

[B37] Gwilt K. B. Olliffe N. Hoffing R. Westmoreland S. Schueler A. Miller G. (2019). P132 dextran sulfate sodium-induced colitis IS attenuated IN trace amine associated receptor 1 knockout mice. Inflamm. Bowel Dis. 25 (Suppl. 1), S63–S63. 10.1093/ibd/izy393.150

[B38] Han X. (2016). Lipidomics for studying metabolism. Nat. Rev. Endocrinol. 12 (11), 668–679. 10.1038/nrendo.2016.98 27469345

[B39] Hashimoto D. Asakura S. Matsuoka K.-i. Sakoda Y. Koyama M. Aoyama K. (2007). FTY720 enhances the activation-induced apoptosis of donor T cells and modulates graft-versus-host disease. Eur. J. Immunol. 37 (1), 271–281. 10.1002/eji.200636123 17154260

[B40] Herrero-Sánchez M. C. Rodríguez-Serrano C. Almeida J. San Segundo L. Inogés S. Santos-Briz Á. (2016). Targeting of PI3K/AKT/mTOR pathway to inhibit T cell activation and prevent graft-versus-host disease development. J. Hematol. Oncol. 9 (1), 113 10.1186/s13045-016-0343-5 27765055 PMC5072323

[B41] Hongo D. Bryson J. S. Kaplan A. M. Cohen D. A. (2004). Endogenous nitric oxide protects against T cell-dependent lethality during graft-versus-host disease and idiopathic pneumonia syndrome. J. Immunol. 173 (3), 1744–1756. 10.4049/jimmunol.173.3.1744 15265904

[B42] Jasperson L. K. Bucher C. Panoskaltsis-Mortari A. Taylor P. A. Mellor A. L. Munn D. H. (2008). Indoleamine 2,3-dioxygenase is a critical regulator of acute graft-versus-host disease lethality. Blood 111 (6), 3257–3265. 10.1182/blood-2007-06-096081 18077788 PMC2265461

[B43] Johnson C. H. Ivanisevic J. Siuzdak G. (2016). Metabolomics: beyond biomarkers and towards mechanisms. Nat. Rev. Mol. Cell Biol. 17 (7), 451–459. 10.1038/nrm.2016.25 26979502 PMC5729912

[B44] Kalaeva E. Kalaev V. Efimova K. Chernitskiy A. Safonov V. (2019). Protein metabolic changes and nucleolus organizer regions activity in the lymphocytes of neonatal calves during the development of respiratory diseases. Vet. World 12 (10), 1657–1667. 10.14202/vetworld.2019.1657-1667 31849429 PMC6868248

[B45] Kang J. Zhu L. Lu J. Zhang X. (2015). Application of metabolomics in autoimmune diseases: insight into biomarkers and pathology. J. Neuroimmunol. 279, 25–32. 10.1016/j.jneuroim.2015.01.001 25669996

[B46] Kapoor S. R. Filer A. Fitzpatrick M. A. Fisher B. A. Taylor P. C. Buckley C. D. (2013). Metabolic profiling predicts response to anti-tumor necrosis factor α therapy in patients with rheumatoid arthritis. Arthritis Rheum. 65 (6), 1448–1456. 10.1002/art.37921 23460124 PMC3715109

[B47] Kim Y.-M. Sachs T. Asavaroengchai W. Bronson R. Sykes M. (2003). Graft-versus-host disease can be separated from graft-versus-lymphoma effects by control of lymphocyte trafficking with FTY720. J. Clin. Invest. 111 (5), 659–669. 10.1172/jci200316950 12618520 PMC151899

[B48] Kominsky D. J. Campbell E. L. Colgan S. P. (2010). Metabolic shifts in immunity and inflammation. J. Immunol. 184 (8), 4062–4068. 10.4049/jimmunol.0903002 20368286 PMC4077461

[B49] Korbecki J. Bajdak-Rusinek K. (2019). The effect of palmitic acid on inflammatory response in macrophages: an overview of molecular mechanisms. Inflamm. Res. 68 (11), 915–932. 10.1007/s00011-019-01273-5 31363792 PMC6813288

[B50] Kumar S. Mohammadpour H. Cao X. (2017). Targeting cytokines in GVHD therapy. J Immunol. Res. Ther. 2 (1), 90–99. Published online June 28, 2017. PMCID: PMC5557058 NIHMSID: NIHMS891689 PMID: 28819653 28819653 PMC5557058

[B51] Kumar S. Dikshit M. (2019). Metabolic insight of neutrophils in health and disease. Front. Immunol. 10, 2099–2099. 10.3389/fimmu.2019.02099 31616403 PMC6764236

[B52] Lampropoulou V. Sergushichev A. Bambouskova M. Nair S. Vincent E. E. Loginicheva E. (2016). Itaconate links inhibition of succinate dehydrogenase with macrophage metabolic remodeling and regulation of inflammation. Cell Metabol. 24 (1), 158–166. 10.1016/j.cmet.2016.06.004 PMC5108454 27374498

[B53] Lee J. H. Park E. Jin H. J. Lee Y. Choi S. J. Lee G. W. (2017). Anti-inflammatory and anti-genotoxic activity of branched chain amino acids (BCAA) in lipopolysaccharide (LPS) stimulated RAW 264.7 macrophages. Food Sci. Biotechnol. 26 (5), 1371–1377. 10.1007/s10068-017-0165-4 30263672 PMC6049802

[B54] Lewis R. N. A. H. McElhaney R. N. (2009). The physicochemical properties of cardiolipin bilayers and cardiolipin-containing lipid membranes. Biochim. Biophys. Acta Biomembr. 1788 (10), 2069–2079. 10.1016/j.bbamem.2009.03.014 19328771

[B55] Liabeuf S. Glorieux G. Lenglet A. Diouf M. Schepers E. Desjardins L. (2013). Does P-cresylglucuronide have the same impact on mortality as other protein-bound uremic toxins? PLoS ONE 8 (6), e67168 10.1371/journal.pone.0067168 23826225 PMC3691113

[B56] Liu Y. Huang A. Chen Q. Chen X. Fei Y. Zhao X. (2019). A distinct glycerophospholipid metabolism signature of acute graft versus host disease with predictive value. JCI Insight 4 (16), e129494 10.1172/jci.insight.129494 PMC6777804 31343987

[B57] Marini B. L. Choi S. W. Byersdorfer C. A. Cronin S. Frame D. G. (2015). Treatment of dyslipidemia in allogeneic hematopoietic stem cell transplant patients. Biol. Blood Marrow Transplant. 21 (5), 809–820. 10.1016/j.bbmt.2014.10.027 25459644 PMC4408224

[B58] Mathewson N. D. Jenq R. Mathew A. V. Koenigsknecht M. Hanash A. Toubai T. (2016). Gut microbiome-derived metabolites modulate intestinal epithelial cell damage and mitigate graft-versus-host disease. Nat. Immunol. 17 (5), 505–513. 10.1038/ni.3400 26998764 PMC4836986

[B59] McNamee E. N. Korns Johnson D. Homann D. Clambey E. T. (2013). Hypoxia and hypoxia-inducible factors as regulators of T cell development, differentiation, and function. Immunol. Res. 55 (1–3), 58–70. 10.1007/s12026-012-8349-8 22961658 PMC3919451

[B60] Metallo C. M. Gameiro C. M. Bell P. A. Mattaini E. L. Yang K. R. Hiller K. (2011). Reductive glutamine metabolism by IDH1 mediates lipogenesis under hypoxia. Nature 481 (7381), 380–384. 10.1038/nature10602 22101433 PMC3710581

[B61] Michonneau D. Latis E. Curis E. Dubouchet L. Ramamoorthy S. Ingram B. (2019). Metabolomics analysis of human acute graft-versus-host disease reveals changes in host and microbiota-derived metabolites. Nat. Commun. 10 (1), 5695 10.1038/s41467-019-13498-3 31836702 PMC6910937

[B62] Michonneau D. Latis E. Dubouchet L. Peffault De Latour R. Robin M. Sicre De Fontbrune F. (2018). Metabolomics profiling after allogeneic hematopoietic stem cell transplantation unravels a specific signature in human acute GVHD. Blood 132 (Suppl. 1), 69–69. 10.1182/blood-2018-99-110441

[B63] Mills E. L. Kelly B. O’Neill L. A. J. (2017). Mitochondria are the powerhouses of immunity. Nat. Immunol. 18 (5), 488–498. 10.1038/ni.3704 28418387

[B64] Munn D. H. Mellor A. L. (2013). Indoleamine 2,3 dioxygenase and metabolic control of immune responses. Trends Immunol. 34 (3), 137–143. 10.1016/j.it.2012.10.001 23103127 PMC3594632

[B65] Nguyen H. D. Chatterjee S. Haarberg K. M. K. Wu Y. Bastian D. Heinrichs J. (2016). Metabolic reprogramming of alloantigen-activated T cells after hematopoietic cell transplantation. J. Clin. Invest. 126 (4), 1337–1352. 10.1172/jci82587 26950421 PMC4811142

[B66] Nguyen H. D. Kuril S. Bastian D. Yu X.-Z. (2018). T-cell metabolism in hematopoietic cell transplantation. Front. Immunol. 9, 176 10.3389/fimmu.2018.00176 29479351 PMC5811499

[B67] Paczesny S. (2013). Discovery and validation of graft-versus-host disease biomarkers. Blood 121 (4), 585–594. 10.1182/blood-2012-08-355990 23165480 PMC3557644

[B68] Pandiyan P. Bhaskaran N. Zou M. Schneider E. Jayaraman S. Huehn J. (2019). Microbiome dependent regulation of Tregs and Th17 cells in mucosa. Front. Immunol. 10, 426 10.3389/fimmu.2019.00426 30906299 PMC6419713

[B69] Park M.-J. Lee S.-Y. Moon S.-J. Son H.-J. Lee S.-H. Kim E.-K. (2016). Metformin attenuates graft-versus-host disease via restricting mammalian target of rapamycin/signal transducer and activator of transcription 3 and promoting adenosine monophosphate-activated protein kinase-autophagy for the balance between T helper 17 and Tregs. Transl. Res. 173, 115–130. 10.1016/j.trsl.2016.03.006 27126953

[B70] Pearce E. L. Pearce E. J. (2013). Metabolic pathways in immune cell activation and quiescence. Immunity 38 (4), 633–643. 10.1016/j.immuni.2013.04.005 23601682 PMC3654249

[B71] Poveda J. Sanchez-Niño M. D. Glorieux G. Sanz A. B. Egido J. Vanholder R. (2014). p-cresyl sulphate has pro-inflammatory and cytotoxic actions on human proximal tubular epithelial cells. Nephrol. Dial. Transplant. 29 (1), 56–64. 10.1093/ndt/gft367 24166466

[B72] Raha S. Raud B. Oberdörfer L. Castro C. N. Schreder A. Freitag J. (2016). Disruption of de novo fatty acid synthesis via acetyl‐CoA carboxylase 1 inhibition prevents acute graft‐versus‐host disease. Eur. J. Immunol. 46 (9), 2233–2238. 10.1002/eji.201546152 27338930

[B73] Reikvam H. Grønningsæter I.-S. Mosevoll K. A. Lindås R. Hatfield K. Bruserud Ø. (2017). Patients with treatment-requiring chronic graft versus host disease after allogeneic stem cell transplantation have altered metabolic profiles due to the disease and immunosuppressive therapy: potential implication for biomarkers. Front. Immunol. 8, 1979 10.3389/fimmu.2017.01979 29416533 PMC5787552

[B74] Reikvam H. Hatfield K. Bruserud Ø. (2016). The pretransplant systemic metabolic profile reflects a risk of acute graft versus host disease after allogeneic stem cell transplantation. Metabolomics 12 (1), 12–12. 10.1007/s11306-015-0880-x 27829829 PMC5080330

[B75] Rezende B. M. Athayde R. M. Gonçalves W. A. Resende C. B. Teles de Tolêdo Bernardes P. Perez D. A. (2017). Inhibition of 5-lipoxygenase alleviates graft-versus-host disease. J. Exp. Med. 214 (11), 3399–3415. 10.1084/jem.20170261 28947611 PMC5679175

[B76] Robinson G. A. Waddington K. E. Pineda-Torra I. Jury E. C. (2017). Transcriptional regulation of T-cell lipid metabolism: implications for plasma membrane lipid rafts and T-cell function. Front. Immunol. 8, 1636 10.3389/fimmu.2017.01636 29225604 PMC5705553

[B77] Schepers E. Meert N. Glorieux G. Goeman J. Van der Eycken J. Vanholder R. (2007). P-cresylsulphate, the main *in vivo* metabolite of p-cresol, activates leucocyte free radical production. Nephrol. Dial. Transplant.: Off. Pub. Eur. Dial. Transplant Assoc. Eur. Renal Assoc. 22, 592–596. 10.1093/ndt/gfl584 17040995

[B78] Schieber M. Chandel N. S. (2014). ROS function in redox signaling and oxidative stress. Curr. Biol. 24 (10), R453–R462. 10.1016/j.cub.2014.03.034 24845678 PMC4055301

[B79] Sellers K. Fox M. P. Bousamra M. 2nd Slone S. P. Higashi R. M. Miller D. M. (2015). Pyruvate carboxylase is critical for non-small-cell lung cancer proliferation. J. Clin. Invest. 125 (2), 687–698. 10.1172/jci72873 25607840 PMC4319441

[B80] Serhan C. N. Chiang N. Van Dyke T. E. (2008). Resolving inflammation: dual anti-inflammatory and pro-resolution lipid mediators. Nat. Rev. Immunol. 8 (5), 349–361. 10.1038/nri2294 18437155 PMC2744593

[B81] Sofi M. H. Wu Y. Dai M. Schutt S. D. Daenthanasanmak A. Heinrichs J. L. (2017). Oxidative stress regulates T cell pathogenicity in gvhd. Blood 130 (Suppl. 1), 3168–3168.

[B82] Suh J. H. Kanathezhath B. Shenvi S. Guo H. Zhou A. Tiwana A. (2014). Thiol/redox metabolomic profiling implicates GSH dysregulation in early experimental graft versus host disease (GVHD). PLoS One 9 (2), e88868 10.1371/journal.pone.0088868 24558439 PMC3928313

[B83] Summers S. (2006). Ceramides in insulin resistance and lipotoxicity. Prog. Lipid Res. 45 (1), 42–72. 10.1016/j.plipres.2005.11.002 16445986

[B84] Sun C.-Y. Hsu H.-H. Wu M.-S. (2013). p-Cresol sulfate and indoxyl sulfate induce similar cellular inflammatory gene expressions in cultured proximal renal tubular cells. Nephrol. Dial. Transplant. 28 (1), 70–78. 10.1016/j.plipres.2005.11.002 22610984

[B85] Taniguchi M. Okazaki T. (2014). The role of sphingomyelin and sphingomyelin synthases in cell death, proliferation and migration-from cell and animal models to human disorders. Biochim. Biophys. Acta Mol. Cell Biol. Lipids 1841 (5), 692–703. 10.1016/j.bbalip.2013.12.003 24355909

[B86] Tannahill G. M. Curtis A. M. Adamik J. Palsson-McDermott E. M. McGettrick A. F. Goel G. (2013). Succinate is an inflammatory signal that induces IL-1β through HIF-1α. Nature 496 (7444), 238–242. 10.1038/nature11986 23535595 PMC4031686

[B87] Tijaro-Ovalle N. M. Karantanos T. Wang H.-T. Boussiotis V. A. (2019). Metabolic targets for improvement of allogeneic hematopoietic stem cell transplantation and graft-vs.-host disease. Front. Immunol. 10 (295). 10.3389/fimmu.2019.00295 PMC6411635 30891031

[B88] Tripathy D. Mohanty P. Dhindsa S. Syed T. Ghanim H. Aljada A. (2003). Elevation of free fatty acids induces inflammation and impairs vascular reactivity in healthy subjects. Diabetes 52 (12), 2882–2887. 10.2337/diabetes.52.12.2882 14633847

[B89] Vallance P. Leiper J. (2002). Blocking NO synthesis: how, where and why? Nat. Rev. Drug Discov. 1 (12), 939–950. 10.1038/nrd960 12461516

[B90] Vander Heiden M. G. Cantley L. C. Thompson C. B. (2009). Understanding the Warburg effect: the metabolic requirements of cell proliferation. Science 324 (5930), 1029–1033. 10.1126/science.1160809 19460998 PMC2849637

[B91] Viola A. Munari F. Sánchez-Rodríguez R. Scolaro T. Castegna A. (2019). The metabolic signature of macrophage responses. Front. Immunol. 10 (1462). 10.3389/fimmu.2019.01462 PMC6618143 31333642

[B92] Wang R. Dillon C. P. Shi L. Z. Milasta S. Carter R. Finkelstein D. (2011). The transcription factor Myc controls metabolic reprogramming upon T lymphocyte activation. Immunity 35 (6), 871–882. 10.1016/j.immuni.2011.09.021 22195744 PMC3248798

[B93] Wen Q. Yang S. Lyu Z. S Chen Y. H. Han T. T Yu W. (2019). “Regulation of the Elevated T cell glycolysis may alleviate acute graft-versus-host disease post-allotransplant. Blood 134 (Suppl. 1), 871–882. 10.1182/blood-2019-122795.

[B94] Wikoff W. R. Anfora A. T. Liu J. Schultz P. G. Lesley S. A. Peters E. C. (2009). Metabolomics analysis reveals large effects of gut microflora on mammalian blood metabolites. Proc. Natl. Acad. Sci. U.S.A. 106 (10), 3698–3703. 10.1073/pnas.0812874106 19234110 PMC2656143

[B95] Williams N. C. O’Neill L. A. J. (2018). A role for the Krebs cycle intermediate citrate in metabolic reprogramming in innate immunity and inflammation. Front. Immunol. 9, 141 10.3389/fimmu.2018.00141 29459863 PMC5807345

[B96] Windt G. J. W. Pearce E. L. (2012). Metabolic switching and fuel choice during T-cell differentiation and memory development. Immunol. Rev. 249 (1), 27–42. 10.1111/j.1600-065X.2012.01150.x 22889213 PMC3645891

[B97] Wong J. M. W. de Souza R. Kendall C. W. C. Emam A. Jenkins D. J. A. (2006). Colonic health: fermentation and short chain fatty acids. J. Clin. Gastroenterol. 40 (3), 235–243. 10.1097/00004836-200603000-00015 16633129

[B98] Wu W. Shi X. Xu C. (2016). Regulation of T cell signalling by membrane lipids. Nat. Rev. Immunol. 16 (11), 690–701. 10.1038/nri.2016.103 27721483

[B99] Wu X. Xie Y. Wang C. Han Y. Bao X. Ma S. (2018). Prediction of acute GVHD and relapse by metabolic biomarkers after allogeneic hematopoietic stem cell transplantation. JCI Insight 3 (9), e99672 10.1172/jci.insight.99672 PMC6012513 29720575

[B100] Yang K. Shrestha S. Zeng H. Karmaus P. W. F. Neale G. Vogel P. (2013). T cell exit from quiescence and differentiation into Th2 cells depend on Raptor-mTORC1-mediated metabolic reprogramming. Immunity 39 (6), 1043–1056. 10.1016/j.immuni.2013.09.015 24315998 PMC3986063

[B101] Zhenyukh O. Civantos E. Ruiz-Ortega M. Sánchez M. S. Vázquez C. Peiró C. (2017). High concentration of branched-chain amino acids promotes oxidative stress, inflammation and migration of human peripheral blood mononuclear cells via mTORC1 activation. Free Radic. Biol. Med. 104, 165–177. 10.1016/j.freeradbiomed.2017.01.009 28089725

[B102] Zou Y. Chen B. J. (2020). T cell metabolism in graft-versus-host disease. Blood Sci. 2 (1), 16–21. 10.1097/bs9.0000000000000035. PMC8974895 35399863

